# Peripheral tryptophan and serotonin and kynurenine pathways in major depression: A case-control study

**DOI:** 10.1192/j.eurpsy.2021.880

**Published:** 2021-08-13

**Authors:** R. Colle, C. Verstuyft, D. David, P. Chanson, E. Corruble

**Affiliations:** 1 Moods Team, INSERM UMR-1178, Le Kremlin-Bicêtre, France; 2 Endocrinology, INSERM 1185, Le Kremlin-Bicêtre, France

**Keywords:** Major Depression, Peripheral serotonin pathway, Peripheral kynurenine pathway, Peripheral tryptophan

## Abstract

**Introduction:**

The tryptophan pathway along with its two branches of metabolism to serotonin and kynurenine seems to be affected in major depression. In depressed patients, peripheral levels of tryptophan, serotonin, kynurenine and their metabolite remain unclear.

**Objectives:**

Therefore, peripheral tryptophan and metabolites of serotonin and kynurenine were investigated extensively in 173 patients suffering from a current major depressive episode (MDE) and compared to 214 healthy controls (HC).

**Methods:**

Fasting plasma levels of 11 peripheral metabolites were quantified: tryptophan, serotonin pathway (serotonin, its precursor 5-hydroxy-tryptophan and its metabolite the 5-hydroxy-indole acetic acid), and kynurenine pathway (kynurenine and six of its metabolites including anthranilic acid, kynurenic acid, nicotinamide, picolinic acid, xanthurenic acid and 3-hydroxy-anthranilic acid).

**Results:**

60 (34.7%) patients were antidepressant drug free. Tryptophan levels did not differ between MDE patients and HC. Serotonin and its precursor (5-hydroxy-tryptophan) levels were lower in MDE patients than HC. Whereas, its metabolite (5-hydroxy-indole acetic acid) levels were within the standard range. Kynurenine and four of its metabolites (kynurenic acid, nicotinamide, picolinic acid and xanthurenic acid) were lower in MDE patients.

**Conclusions:**

This study uses the largest ever sample of MDE patients, with an extensive assessment of peripheral tryptophan metabolism in plasma. These findings provide new insights into the peripheral signature of MDE. The reasons for these changes should be further investigated. These results might suggest a better stratification of patients and different therapeutic strategies therapeutic strategies.

